# Life in a London Hospital. II

**Published:** 1890-08-09

**Authors:** 


### Life in a London Hospital.—II.

*(Continued from page 255.)*

At half-past twelve we had dinner, the "Sister" of the ward presiding at the table, and the staff-nurse, helped by some of the convalescent patients, carrying round each plate.

Then the ward was again swept, and at two, silence was obligatory for an hour to allow the patients to sleep, if they wished. Between three and half-past four, doctors were continually visiting the ward, and after that we had tea. Prayers were read at six, followed by one or two hymns, the choice of the hymn being left entirely to the patients. The two favourites were:

"Not mine, but Thine, Oh Lord."

and Keble's beautiful evening hymn,

"Sun of my soul, thou Saviour dear."

All the patients joined most readily in the singing, and, indeed, they seemed sorry when the hymns had come to an end.

In the evening the staff-nurse was always on duty. She was a very great favourite in the ward, one and all loved her. "Oh! ask Nurse Rayner if ever you want anything," the patients used to tell me. The sight alone of her sweet face did one good. She looked so thoroughly contented, as she hurried about from bed to bed, and was ever ready with a sympathetic word. Whenever she was attending to us, she had a smile on her face, but I have watched her now and then standing still, and seen such a sad troubled look come across the dark blue eyes that seemed to say, she had suddenly found a minute or so in which she could think about some special grief of her own. At eight the lights were lowered for the night, and we were supposed to go to sleep. At first I slept well, but afterwards I found the nights very wearisome and long. How I wondered if the morning would ever come, as I listened to Big Ben chiming forth each quarter of an hour.

The chimes are beautiful, they are as follows:

"Lord, through this hour,
Be Thou my guide,
Helped by Thy power
No foot shall slide."

It was such a comfort to watch the tall slight figure of the night-nurse, as she passed backwards and forwards, attending to the wants of every patient, and it was a relief to be able to call "Nurse," and to have her just for an instant, bending over your bed to smooth your pillows, and to give you all the relief that was in her power.

The night nurse was quite as popular among the patients as Nurse Rayner was, and we used to see a great deal of her too, as she was on duty from ten in the evening till ten the next morning. She was so cheerful and sympathetic, and devoted to the children. Many a time I have seen her lift a little one out of bed and kiss her tenderly as she held the child in her arms. How these nurses are to be envied! The whole of their lives are like one long day of love and self-sacrifice, and when their time of rest comes and God sends for them, they will not have to go to Him empty-handed as so many of us will.

I have already mentioned "Jennie," my little friend who offered me the jam. One visiting afternoon she was seated on my locker just before the visitors arrived. I told her I was expecting an auntie to see me, "Oh, I've got an auntie too," she exclaimed, "she keeps a shop for eels." Then, seeing that I looked rather puzzled as to what she meant by an eel-shop, she began to explain, "Why, you know what eels are, don't you? Stewed eels, you know; lovely things stewed eels are." And then she continued, "Auntie has eels in one winder and meat-pies in the other, and you know what meat-pies are, don't you?" This last question was asked in a most emphatic manner, as much as to say that if I did not know what meat-pies were I was a fearful ignoramus.

After my operation, No. 26, a girl who had lost the use of one of her hands, sat by my bed for hours, bathing my head, talking to me all the while in such a quiet soothing voice. And another morning, No. 4, a young married woman opposite, saw me trying to reach something out of my locker, and when she saw I could not manage it she came across to me immediately. I had been crying from the effects of a bad night, and although I was a perfect stranger to her she took me in her arms in such a motherly way, kissing me several times. "You mustn't cry, my darling," she said, "you mustn't; why, very soon you will be going home to your friends, and think how glad they will be to have you." How I loved that woman for her comforting words!

These are but a few of the many kindnesses I received from my fellow-patients, but I first learnt to know their true worth, simply from seeing their kindnesses one to another.

The children used to call me Mrs. 28, and No. 3 was very anxious to know my age. I told her I was twenty-four, so she exclaimed, "What, twenty-four, and not married!" The little ones would come with their pennies, to buy a pennyworth of envelopes or note-paper, and would I sell them a stamp to write to mother with? It was a pleasure to see their little faces light up when I shook my head over the proffered penny. And my scent, too, was in great demand. They would stand round my bed to know if they might have just a smell out of the bottle, but No. 3 was older and wiser than some of the other children, and she invariably produced a little bottle out of her pocket, which I replenished periodically, and another time she came to tell she had been washing out her handkerchief, and would I give her some scent to put on it. I fancy No. 3 will grow up with a good eye to business, as she made a very clever bargain one day with another child, exchanging a little green frog for a small white prayer book.

And now before closing these few pages, I should like to make an appeal in behalf of those I have left behind me, still lying ill and suffering under the hand of God. Will some of my readers who have large gardens and flowers, and vegetables in abundance to spare, sometimes think of those who are suffering, and send a hamper to the hospitals? At Christmas and Eastertide, flowers are especially wanted, as the nurses take such trouble to make the wards look unusually bright at those festive seasons. And old magazines are always acceptable, and are of great use in amusing the patients, also books and Christmas cards for the children.

